# Profil épidémiologique des entérobactéries uropathogènes productrices de bêta-lactamases à spectre élargi

**DOI:** 10.11604/pamj.2017.28.29.11402

**Published:** 2017-09-13

**Authors:** Mohammed Sbiti, khalid Lahmadi, Lhoussaine louzi

**Affiliations:** 1 Service de Microbiologie, Hôpital Militaire Moulay Ismail de Meknès, Maroc; 2 Faculté de Médecine et de Pharmacie, Université Mohammed Ben Abdellah, Fès, Maroc; 3 Pôle de Biologie Médicale, Hôpital Militaire Moulay Ismail Meknès, Maroc; 4 Faculté de Médecine et de Pharmacie, Université Mohammed V, Souissi-Rabat, Maroc

**Keywords:** Infections urinaires, ECBU, entérobactéries, BLSE, résistance aux antibiotiques, Urinary tract infections, CBEU, enterobacteria, ESBL, antibiotics resistance

## Abstract

Les infections urinaires à entérobactéries productrices de bêtalactamases à spectre élargi (E-BLSE) constituent un risque infectieux, un enjeu thérapeutique de taille et peuvent même conduire dans certains cas à des impasses du fait de leur multi-résistance aux antibiotiques. Le but de ce travail est de préciser le profil épidémiologique des (E-BLSE) uropathogènes et décrire leur niveau actuel de résistance aux antibiotiques pour une meilleure prise en charge des patients selon les données locales. Il s'agit d'une étude rétrospective sur une période de trois ans (du 1^er^ janvier 2013 au 31 décembre 2015) concernant toutes les souches d'E-BLSE isolées de tous les ECBU traités au laboratoire de microbiologie de à l'Hôpital Militaire Moulay Ismail de Meknès. La culture a été faite selon les techniques usuelles, et l'antibiogramme a été réalisé par méthode de disque diffusion sous gélose Muller-Hinton selon les recommandations du Comité de l'antibiogramme de la Société française de microbiologie CA-SFM 2013/2014. Cette étude a permis de noter une importante prévalence globale d'isolement des E-BLSE (12.2%), particulièrement chez les patients hospitalisés (54.8%) dont la plus grande prévalence (72%) a été enregistrée dans le service d'urologie. Parmi ces E-BLSE Escherichia coli constitue la majorité (61%) des isolats, cependant au sein de la même espèce Klebsiella pneumoniae est le plus producteur de BLSE (25.8%). L'étude de l'antibioresistance des E-BLSE durant ces trois ans a mis en évidence des co-résistances à la ciprofloxacine (92.5%), au sulfametoxazole-trimethoprime (88,4%), à la gentamycine (67,2%). Globalement nos résultats sont en accord avec les données des autres pays méditerranéens exception faite pour l'amikacine dont la résistance est très basse (6.1%) dans notre étude. Cette étude a montré que la prévalence des E-BLSE en milieu hospitalier est importante et que sa diffusion en milieu communautaire est un fait préoccupant. Ces E-BLSE sont généralement résistantes aux antibiotiques, notamment des aux molécules utiles en urologie.

## Abstract (en)

Urinary tract infections due to enterobacteria producing extended spectrum beta-lactamases (ESBL-E) constitute a infectious risk, a major therapeutic challenge and can even lead, in some cases, to a deadlock beacuse of their multi-resistance to antibiotics. This study aimed to determine the epidemiological profile of uropathogenic ESBL-E and to describe their current level of resistance to antibiotics for a better patient management according to local data. We conducted a retrospective study of all ESBL-E strains isolated from all cytobacteriogical testing of urine (CBEU) treated in the Microbiology Laboratory at the Military Hospital of Moulay Ismail, Meknes over a period of three years (from 1 January 2013 to 31 December 2015). Culture was performed according to conventional techniques and antibiogram was performed using Mueller-Hinten agar disk diffusion susceptibility test according to the recommendations from the Committee on Antimicrobial Susceptibility of the French Society for Microbiology (CA-SFM), year 2013/2014. This study allowed to report a significant overall prevalence of isolation of ESBL-E (12.2%), particularly among hospitalized patients (54.8%). The greater prevalence (72%) was registered in the Department of Urology. Among these, ESBL-E Escherichia coli constitutes the majority (61%) of the isolates, however Klebsiella pneumoniae is the major ESBL producer (25.8%) within the same species. The analysis of ESBL-E antibioresistance conducted during these three years revealed some co-resistances to ciprofloxacin (92.5%), sulfametoxazole-trimethoprim (88.4%), gentamycin (67.2%). Globally, our results are compliant with the data from the other Mediterranean countries, except for amikacin whose resistance was very low (6.1%) in our study. This study shows that the prevalence of ESBL-E in hospital is high and that its diffusion in community setting is a matter of concern. These ESBL-E are generally resistant to antibiotics, including molecules useful in urology.

## Introduction

L'infection urinaire (IU) représente, partout dans le monde, l'un des principaux motifs de consultation, d'explorations microbiologiques et l'utilisation intensive des antibiotiques avec, pour cette dernière, les conséquences sur le coût des soins et la sélection de souches multi résistantes aussi bien en milieu hospitalier qu'en milieu communautaire [1]. La résistance des entérobactéries aux céphalosporines de troisième génération (C3G) est fortement renforcée par l'acquisition des gènes codant les bêta-lactamases à spectre élargi (BLSE). Ces enzymes (TEM, SHV, CTX-M et dérivés) confèrent aux entérobactéries la résistance à l'ensemble des bêtalactamines à l'exception des céphamycines et des carbapénèmes en plus d'une résistance associée à d'autres familles d'antibiotiques [2]. Alors que les entérobactéries productrices de bêtalactamases à spectre élargi (EBLSE) étaient observées essentiellement en milieu hospitalier, la diffusion de ces germes multirésistantes en milieu communautaire est de plus en plus inquiétante. La transmission, principalement plasmidique, des gènes codants pour les BLSE est responsable de leur dissémination rapide et ainsi de l'augmentation de la prévalence des bactéries productrices de BLSE partout dans le monde [3]. Dans notre pays, bien que la maîtrise de la diffusion de ces bactéries multi-résistantes constitue une priorité, peu de données actualisées permettent de définir l'ampleur de ce phénomène au niveau de la région de Meknès. L'objectif de ce travail est de déterminer la prévalence des entérobactéries productrices de BLSE uropathogènes dans notre région et de préciser leurs profils de résistance aux antibiotiques. L'ensemble de ces informations devrait également permettre d'appréhender l'importance du risque de circulation de ces entérobactéries multirésistantes et de formuler des conseils ayant pour but le renforcement de la maitrise de leurs diffusion aussi bien au niveau hospitalier que communautaire à l'échelle régionale.

## Méthodes

**Figure 1:**
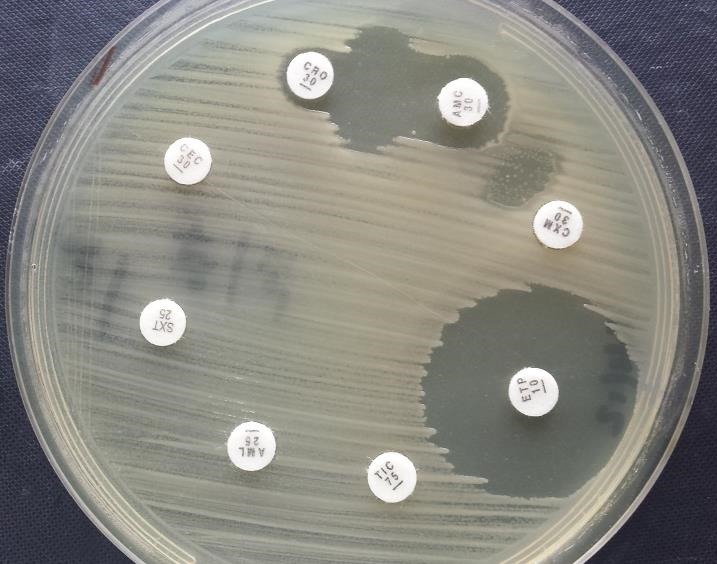
Test de synergie positif: apparition de l’aspect en « bouchon de champagne» entre les 2 disques d’antibiotiques (AMC et CRO)

**Figure 2:**
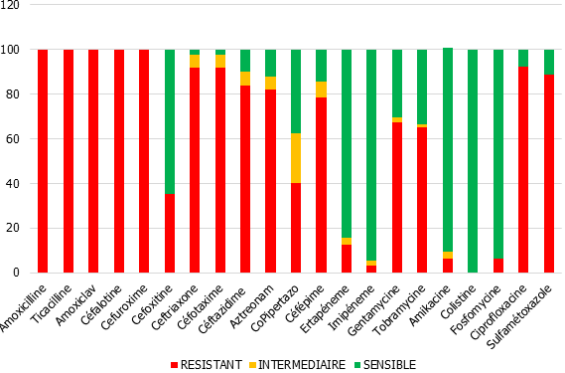
Profil de co-résistance des EBLSE aux différents antibiotiques testés

Il s'agit d'une étude rétrospective à visée épidémiologique sur une période de trois ans (du 1^er^ janvier 2013 au 31 décembre 2015) concernant toutes les souches d'E-BLSE isolées à partir des prélèvements urinaires, a visée diagnostique, adressés au laboratoire de microbiologie de l'hôpital Moulay Ismail de Meknès (HMMIM). Cet hôpital a une capacité de 300 lits et comporte toutes les spécialités et les consultations correspondantes en dehors de la pédiatrie. Les prélèvements urinaires proviennent de patients hospitalisés dans différents services ou accueillis dans le cadre des urgences ou chez des patients consultants en ambulatoire (dispensaires, consultations externes). Les doublons étant systématiquement éliminés. Le recueil des urines dans un flacon stérile spécial, avant toute antibiothérapie; et traité dans les deux heures qui suivent la réception. Dès réception du prélèvement, la conformité flacon-demande d'ECBU est contrôlée et l'aspect macroscopique noté. Chaque urine a fait l'objet d'un examen cytobactériologique urinaire de routine comportant : 1) un examen microscopique en cellule Fast Read^®^ permet de noter les éléments éventuellement présents: leucocytes et les hématies et les autres éléments figurés de l'urine (les cellules épithéliales, les cylindres, cristaux. . .); 2) une uroculture avec dénombrement de germes (bactériurie). Les milieux de cultures utilisés sont la gélose BCP lactosée (systématique) avec en option, la gélose au sang en cas d'observation de cocci en chaînettes ; et le Sabouraud+chloramphénicol en cas de présence de levures. L'interprétation utilisée est classique (critères défini par Kass) et tient compte de paramètres d'infection urinaire: leucocyturie >10^4^//mL; bactériurie en unité formant colonie (UFC) par mL >10^5^/mL sachant que Escherichia coli et Staphylococcus saprophyticus étant considérés comme uropathogènes spécifiques, leur seuil est abaissé à 10 ^3^/UFC/mL. L'identification des bactéries isolées est basée sur l'utilisation de milieu chromogène type Uriselect^®^ de BioRad, les systèmes Api 20 (E, NE, Staph, Strep) de BioMérieux^™^ et les identifications immunologiques par latex agglutination (Staph aureus, Streptocoque B,…). L'étude de la sensibilité aux antibiotiques a été pratiquée par diffusion en milieu gélosé Mueller-Hinton et l'interprétation a été faite selon les recommandations du CA-SFM [4]. La détection des BLSE est effectuée par méthode de synergie entre l'acide clavulanique du disque du co-amoxiclave (AMC) et la céftriaxone (CRO) ou bien à la céfotaxime (CTX), caractérisée par une image en « bouchon de champagne » et signe la présence d'une BLSE (Figure 1). Toute entérobactérie catégorisée BLSE positive est confirmée avec d'autres disques: ceftazidime (CAZ), aztréonam (ATM), céfépime (FEP) et testée vis-à-vis des carbapénèmes. En cas de résistance à l'ertapénème (ETP), un test de Hodge effectué à l'aide d'un disque d'imipénème (IPM) (Figure 2).

## Résultats

Durant une période de trois ans, au niveau du laboratoire de bactériologie de l'HMMIM, nous avons réalisé ECBU un total de 19400 ECBU dont 2522 qui répondent aux critères classiques d'infection urinaire, soit 13,3 % de positivité. Ces prélèvements concernaient essentiellement des patients consultant à titre externe (75,8 % n = 1912). L'analyse des isolats montre les entérobactéries ont constitué 80,5 % des isolats d'urines positives, l'espèce Escherichia coli a dominé l'étiologie des infections urinaires avec un taux de 73% des entérobactéries recensées. La répartition selon le sexe montre une prédominance féminine avec sexe ratio homme/femme de 1.76. L'étude des tranches d'âge montre une nette prédominance des E-BLSE isolées des urines chez les plus de 55 ans qui représentent 72% des cas. La production de BLSE est observée dans 262 cas parmi les 2144 souches d'entérobactéries soit une prévalence moyenne de 12.2%. Les fréquences d'isolement des souches d'entérobactéries productrices de BLSE est passé de 10,25% en 2013 à 13,77% en 2015. Les E-BLSE hospitalière ont représenté 54.8% de l'ensemble des E-BLSE isolées contre 5.2% en ambulatoire. Les services d'hospitalisations les plus concernés étaient: l'urologie 72%, la réanimation 12% et de la chirurgie viscérale 9%. La répartition des EBLSE montre une prédominance des E. coli réalisant environ 63% des cas; suivi de Klebsiella spp 32,8%. La fréquence d'isolement des BLSE au sein de chaque espèce d'entérobactérie montre une capacité de production de BLSE plus importante chez Klebsiella pneumoniae avec 25.8%, suivie de Klebsiella oxytoca avec 20%, d'Enterobacter cloacae avec 11,4% et d'Escherichia coli avec 10,5%, par contre aucune souche de Proteus spp ou de Citrobacter productrice de BLSE n'a été isolée (Tableau 1). Concernant la résistance aux antibiotiques chez les EBLSE, l'analyse des cas a montré une résistance considérable à la combinaison sulfametoxazole / trimethoprime ou cotrimoxazole (88,4%) et à la ciprofloxacine (92.5%). La résistance à la gentamicine chez les E-BLSE a atteint (67,2%). Ces souches ont également monté une résistance de l'ordre de 3.4% et 5,4% vis-à-vis de l'imipenème et de la fosfomycine. Par ailleurs, Ces souches BLSE gardaient une bonne sensibilité à l'Amikacine (6.1% de résistance) (Figure 2 ). Ce qui en fait l'aminoside de choix en cas de nécessité d'association d'antibiotiques pour le traitement.

**Tableau 1:** Répartition selon le genre bactérien des entérobactéries BLSE et la fréquence des EBLSE au sein de leurs espèces bactérienne

Espèce d'entérobactéries	Entérobactérie		E-BLSE		Fréquenced'isolement
	n	%	n	%	
Escherichia coli	1565	73 %	165	63%	10.5 %
Klebsiella oxytoca	225	10,5 %	45	17.2 %	20 %
Klebsiella pneumoniae	159	7,4 %	41	15.6%	25.8 %
Enterobacter spp	105	4,9 %	12	4.6 %	11,4 %
Proteus mirabilis	38	1,8 %	0	-	-
Morganella morganii	16	0,7 %	0	-	-
Providencia spp	12	0,5 %	0	-	-
Citrobacter freundii	9	0,4 %	0	-	-
Autres Entérobactéries	15	0,69%	0	-	-
**Total**	2144	100 %	262		12.2%

## Discussion

Depuis leur première mise en évidence en 1983, les entérobactéries productrices de bêtalactamases à spectre étendu (EBLSE) ont largement diffusé dans le monde avec des fréquences d'isolement variables même d'un service à l'autre au sein de la même institution hospitalière. Les EBLSE aujourd'hui sont des BMR majoritaires qui sont à l'origine d'infections potentiellement sévères et de prescriptions d'antibiotiques à large spectre, qui menacent l'activité future des molécules de dernière ligne. Leur implication dans les infections urinaires (IU) nosocomiales, mais également dans les IU communautaires, constitue un réel problème de santé publique [1]. Le profil des données bactériologiques locales et actualisées sont indispensables pour l'application efficace des nouveaux consensus de la prise en charge de cette pathologie où il s'agit en particulier de prescrire une antibiothérapie de première intention efficace contre les bactéries uro-pathogènes. Notre étude à objectivé une prévalence globale d'isolement des EBLSE de 12.2%. Ce taux est supérieur à celui enregistré par Lahlou et al. (9%) entre 2006 et 2008 sur le même sujet dans cet hôpital [5] témoignant d'une augmentation de la fréquence des EBLSE dans notre formation ou bien d'une plus grande efficacité du dépistage. Ce taux reste néanmoins proche à la fréquence retrouvée dans les études réalisées en 2010 à Rabat (17.5%) [6] et en 2012 à Marrakech (13%) [7]. Des fréquences similaires (dépassant 10 %) ont été enregistrées en Grèce, Turquie, Italie et Portugal [8]. En France et sur l'île de la Réunion, la fréquence d'isolement des E-BLSE était de 5,8% en (2006/2007) [9]. Il est cependant plus important que celui rapporté en Allemagne ou en Grande-Bretagne avec des taux respectifs 2,6%, 2% [10], mais demeure moins important que celui rapporté en Algérie (37,1%) en 2011 et en Tunisie (30.8%) en 2010 [11,12].

D'après les résultats de notre étude, 95,8 % des EBLSE recensées étaient des souches de d´E. Coli et Klebsiella spp. Cela concorde avec les résultats de plusieurs études qui ont mis en évidence que ces deux espèces étaient les plus fréquemment responsables de la production des BLSE [5, 7, 8,13]. Par ailleurs Klebsiella pneumoniae reste l'entérobactérie la plus pourvoyeuse de BLSE au sein de son genre avec une prévalence d'expression de 25.8%. Pour Ben Haj Khalifa et Khedher, Klebsiella spp produisait des BLSE dans 20,2% des cas [14]. Cependant, certains auteurs relatent un déclin de cette dominance en faveur d'E. Coli [15]. Presque 75% de nos patients sont âgés de plus de 55 ans, de nombreuses études confirmant que parmi les facteurs de risque d'ITU par une bactérie multi-résistante (BMR) dont les EBLSE, figure un âge avancé, généralement supérieur à 65 ans [16]. La prédominance masculine des infections urinaires à EBLSE s comme dans notre travail reste controversée, alors que des études l'ont confirmées, d'autres ont rapporté une prédominance féminine [15]. Ces différences peuvent refléter les disparités régionales dans les pratiques de prescription d'antibiotiques liées au sexe, la nature de recrutement des patients dans un hôpital, et également peut être le résultat de biais méthodologiques comme les critères d'inclusions (chirurgie prostatique, …). Selon les résultats de notre étude, les EBLSE étaient majoritairement du type nosocomial (54,8%); ce qui rejoint les données de la littérature issue de différents pays [17] ; Une fréquence importante des EBLSE a été notée dans les services de chirurgie surtout l'urologie d'après notre travail (72%), taux important confirmes dans tous les études en Algérie (40%), au Maroc (35%) en France (23%). Dans notre étude, on a noté une proportion des EBLSE de 12% de patients hospitalisés en réanimation. Ce taux reste moins important par rapport aux données de la littérature celui en Algérie (47,33%), à Rabat (28,2%), ou en France (31%) [6, 9, 11]. Cette prévalence accrue des résistances au niveau des services d'urologie et de réanimation peut être expliquée par la nature des échantillons de patients étudiés et confirme la notion de service à risque où plusieurs facteurs se réunissent pour accentuer ce constat: long séjour hospitalier, gestes et dispositifs invasifs (cathéters, sondes, intubation, …), l'exposition préalable aux antibiotiques parfois administrés en long durée. Autres facteurs divers comme la malnutrition, l'immunodépression, l'hémodialyse, la nutrition parentérale exclusive ou une hospitalisation antérieure peuvent intervenir [18].

L'étude de l'antibio-résistance des E-BLSE uropathogènes isolées au niveau de notre laboratoire a mis en évidence des taux de co-résistance élevés pour la ciprofloxacine (92.5%), la gentamycine (67,2%), tobramycine (65,2%) et le sulfaméthoxazole-triméthoprime (88,4%). Ces taux de co-résistances enregistrés sont similaires à ceux rapportés dans les résultats publiés au Maroc en 2009 à Meknès, en 2012 à Rabat et en 2014 à Marrakech (tableau 2) [5, 6,7]; Ces niveaux de résistance obtenus sont inquiétants et alarmants. Cette situation est la conséquence de la pression de sélection due à la prescription démesurée et l'usage parfois abusif des antibiotiques à large spectre aussi bien en milieu hospitalier qu'en milieu communautaire (délivrance officinale sans ordonnance, automédication, échantillon gratuit, …), sans oublier l'impact de l'alimentation peu contrôlée et où de plus en plus d'antibiotiques sont utilisés en agriculture et dans l'élevage. Le déterminisme plasmidique prédominant de ces résistances acquises favorise, par ailleurs, leur dissémination. Cette importante co-résistance des E-BLSE limite fortement l'arsenal thérapeutique et accroit le risque d'impasse en matière de traitement [19]. Le taux élevé de co-résistance des E-BLSE aux quinolones dans le monde entier compromet l'utilisation de cette classe d'anti-infectieux très utilisée en pratique quotidienne. Ceci peut être essentiellement expliqué ; d'abord par l'utilisation massive de ces antibiotiques pour traiter en 1er intention les infections urinaires sans documentation préalable. Et en second lieu par l'émergence récente de 3 mécanismes de résistance plasmidique aux fluoroquinolones qui sont : le gène Quinolone résistance « Qnr », les gènes codant respectivement pour une N-acétyltransférase, ACC-(6')-Ibcr et les gènes codant pour la pompe d'efflux QepA [20]. Leur association à d'autres déterminants de résistance, essentiellement bêtalactamines et aminosides, favoriserait la co-sélection de la résistance aux fluoroquinolones. Le pourcentage des EBLSE résistantes à la gentamycine (67,2%) était supérieur à ceux résistantes à l'amikacine (6.1%) qui est très bas en comparaison à d'autres études et reste l'aminoside le plus efficace contre les EBLSE. Ainsi, la sensibilité des EBLSE aux aminosides varie en fonction des études et donc des mécanismes impliquées [5, 7, 21].

**Tableau 2:** Comparaison des pourcentages de résistances des EBLSE de certains antibiotiques avec d’autres études

Etude	Pays	Prélèvements	Ciprofloxacine	Amikacine	Gentamycine	Fosfomycine	Imipénème
Ben Haj Khalifa A. et al (2009) (14)	Tunisie	Urines	67.5 %	10 %	92.5%	17.5%	0%
Fouquet .M et al (2005-2009) (13)	France	Divers	74 %	-	41%	-	0%
N.S.M Hailaji et al (2016) (24)	Mauritanie	Urines	33.6 %	-	19.5%	21.2%	0%
Zohreh. A et al (2007) (25)	Iran	Urines	35 %	10%	33.5%	50%	0%
EL Bouamri .M.C. et al (2008-2012) (7)	Maroc (Marrakech)	Urines	82%	51%	74%	13%	10%
Romli et al (2010) (6)	Maroc (Rabat)	Urines	76%	26 %	86%	20%	0%
Lahlou. A et al (2006-2008) (5)	Maroc (Meknès)	Urines	80%	60 %	95%	-	0%
Notre série (2013-2015)	Maroc (Meknès)	Urines	92.7%	6.1 %	62%	5.4%	3.4%

Dans notre étude, la fosfomycine a été active contre les E-BLSE à hauteur de 92.3%. La fosfomycine a montré une activité in vitro prometteuse contre les EBLSE urinaires multirésistants. Cette molécule garde une bonne activité malgré sa forte recommandation dans le traitement des cystites aiguës non compliquées en raison de sa faible prescription [22]. Le problème au Maroc, c'est sa disponibilité fluctuante dans le marché et l'absence de forme injectable à usage hospitalier contre les infections nosocomiales à EBLSE. L'importante résistance des E-BLSE à de nombreuses familles d'antibiotiques réduit considérablement les options thérapeutiques et entretient une hausse continue de la prescription des carbapénèmes qui possèdent une très bonne activité contre les E-BLSE [23, 24]. Sur trois ans, 3.4% des E-BLSE uropathogènes isolées dans notre laboratoire étaient résistantes à l'imipénème, l'ensemble de ces souches appartenaient à l'espèce K. pneumoniae. Ce qui témoigne de l'émergence de souches associant BLSE et imperméabilité aux carbapénèmes ou bien coproduction de BLSE et de carbapénèmases montrant ainsi un phénotype de « panrésistance » aux bêtalactamines [25]. De ce fait, l'utilisation rationnelle des carbapénèmes s'impose car ils représentent l'outil thérapeutique de dernière ligne pour le traitement des infections par bacilles à Gram négatif producteurs de BLSE [26]. Les nouvelles recommandations de la CASFM-EUCAST pour l'interprétation des antibiogrammes des souches productrices de BLSE ont déclaré les C3G et C4G pleinement actif après la détermination de leurs concentrations minimales inhibitrices [4]. Parmi les molécules substitutives des carbapénèmes proposées pour le traitement des infections par E-BLSE, on peut citer les C3G, les C4G, les céphamycines (Cefoxitine, leflomoxef), l'association-lactamine-inhibiteur de bêta-lactamases (Piperacilline/tazobactam), la pivmecillinam, la témocilline, la nitrofurantoïne, et la tigécycline [27]. Des nouvelles molécules ou associations ont atteint les phases II ou III de développement clinique pour le traitement des infections à entérobactéries productrices de BLSE, dont des associations bêta-lactamine-inhibiteur de bêta-lactamases (ceftolozane/ tazobactam, ceftazidime/avibactam, ceftaroline/avibactam), 2 inhibiteurs de la synthèse protéique (plazomicine et eravacycline) et une molécule avec un mode d'action original (brilacidine) [28]. Malgré le rôle important que peuvent jouer ces molécules dans le traitement des infections urinaires à E-BLSE, peu de données cliniques sont disponibles et donc l'activité de ces molécules reste sous-évaluée.

## Conclusion

Les EBLSE dans les infections urinaires constituent, dans notre établissement, un risque infectieux croissant avec des niveaux élevés de résistance aux antibiotiques aussi bien à l'hôpital qu'en médecine de ville; et l'émergence de la résistance aux molécules d'ultime recours en thérapeutique antibactérienne, à savoir, les carbapénèmes. Ils peuvent conduire, dans nombre de cas à une impasse thérapeutique. Ce phénomène de multi-résistance aux antibiotiques est un problème majeur de santé publique au Maroc, inquiétant et alarmant du fait des risques potentiels (morbimortalite augmentée, surcoûts économiques et installation de bactéries hautement résistantes dans les services hospitaliers). Une meilleure maîtrise en termes de respect strict des mesures d'hygiène, l'isolement des porteurs, l'utilisation raisonnée des antibiotiques et définir les stratégies thérapeutiques et prophylactiques adaptées à l'épidémiologie locale sont les actions clés pour ralentir leur émergence et leur dissémination. Par ailleurs, le respect des règles de bonne pratique officinale pour la délivrance des médicaments, le rôle majeur que doivent remplir les pharmaciens de ville comme conseillers en antibiothérapie sont autant d'éléments à promouvoir dans le contrôle de la diffusion de la multi-résistance dans la communauté. La diffusion intra- et extrahospitalière des EBLSE dans les infections urinaires impose à tous les établissements de soins de surveiller toutes les EBLSE. Malgré tout, d'autres études sont nécessaires pour déterminer si nous ne sommes pas là en présence de l'émergence des EBLSE et d'y associer les paramètres cliniques, thérapeutiques et la confrontation avec l'outil moléculaire permettant la classification et le typage des BLSE incriminées, ainsi que l'identification des déterminants génétiques responsables de ces résistances.

### Etat des connaissances actuelle sur le sujet

- Le Maroc fait partie des zones d'endémicité élevée en entérobactéries productrices de bêtalactamases à spectre élargi (E-BLSE);
- Les hôpitaux constituent un véritable réservoir ces E-BLSE;
- Confèrent aux entérobactéries la résistance à l'ensemble des bêtalactamines et de en plus d'une résistance associée à d'autres familles d'antibiotiques, à l'exception des carbapénèmes.

### Contribution de notre étude à la connaissance

- Cette étude a permis de noter une importante prévalence globale d'isolement des E-BLSE (12.2%), particulièrement chez les patients hospitalisés;
- Un taux de co-résistance élevés, aux molécules utiles dans les infections urinaires, à la ciprofloxacine (92.5%), ceftriaxone (97,1%), au sulfametoxazole-trimethoprime (88,4%), à la gentamycine (67,2%);
- Une sensibilité importante des E-BLSE dans notre formation à la colistine (100%) à l'amikacine (91.4%), à la fosfomycine (92,3%).

## Conflits d’intérêts

Les auteurs ne déclarent aucun conflit d'intérêts.
